# MEGANE investigations of Phobos and the Small Body Mapping Tool

**DOI:** 10.1186/s40623-021-01509-x

**Published:** 2021-12-13

**Authors:** Nancy L. Chabot, Patrick N. Peplowski, Carolyn M. Ernst, Hari Nair, Michael Lucks, R. Josh Steele, David J. Lawrence

**Affiliations:** Johns Hopkins University Applied Physics Laboratory, 11100 Johns Hopkins Rd, Laurel, MD 20723 USA

**Keywords:** Phobos, MMX, MEGANE, Gamma-ray spectroscopy, Neutron spectroscopy, Martian moons

## Abstract

The MEGANE instrument onboard the MMX mission will acquire gamma-ray and neutron spectroscopy data of Phobos to determine the elemental composition of the martian moon and provide key constraints on its origin. To produce accurate compositional results, the irregular shape of Phobos and its proximity to Mars must be taken into account during the analysis of MEGANE data. The MEGANE team is adapting the Small Body Mapping Tool (SBMT) to handle gamma-ray and neutron spectroscopy investigations, building on the demonstrated record of success of the SBMT being applied to scientific investigations on other spacecraft missions of irregularly shaped bodies. This is the first application of the SBMT to a gamma-ray and neutron spectroscopy dataset, and the native, three-dimensional foundation of the SBMT is well suited to MEGANE’s needs. In addition, the SBMT will enable comparisons between the MEGANE datasets and other datasets of the martian moons, including data from previous spacecraft missions and MMX’s multi-instrument suite.

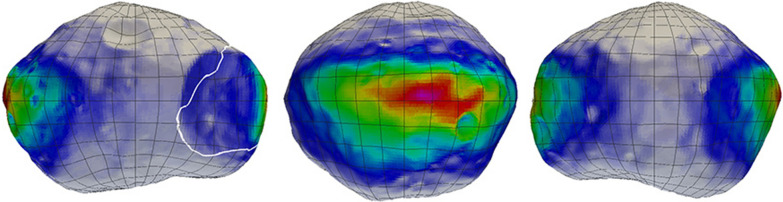

## Introduction

Although the two moons of Mars, Phobos and Deimos, were discovered over a century ago, much about their origins, histories, and natures remain largely a mystery. The Martian Moons eXploration (MMX) mission (Kuramoto et al. 2021), led by the Japan Aerospace Exploration Agency (JAXA), is on track to become the first dedicated mission to explore and sample the martian moons. One of the scientific instruments on the MMX mission is MEGANE (Mars-moon Exploration with GAmma rays and NEutrons), a NASA-funded gamma-ray spectrometer (GRS) and neutron spectrometer (NS) (Lawrence et al. 2019). MEGANE will measure the elemental composition of Phobos, providing crucial data to understand the formation of the martian moons.

For MEGANE, there are two factors about Phobos that need to be properly taken into account for deriving accurate elemental abundances. The first factor is Phobos’ irregular (i.e., non-spherical) shape, which will modify the measured gamma-ray and neutron fluxes in ways different from a more spherical body due to the different angles, altitudes, and viewing geometries created by the large-scale surface topography and the need for MEGANE to collect scientific measurements within an altitude of one Phobos radius or less from the surface (Lawrence et al. 2019). The second factor is Phobos’ close proximity to Mars. Mars blocks the cosmic rays that produce gamma-rays and neutrons, producing a hemispherical asymmetry that will alter the MEGANE measurements. Additionally, Mars-originating gamma rays and neutrons will cause a time-dependent background that needs to be quantified and removed from the measured MEGANE data.

The Small Body Mapping Tool (SBMT) is an interactive three-dimensional visualization and data analysis tool developed at the Johns Hopkins University Applied Physics Laboratory to map and analyze features on irregular shaped solar system bodies (Ernst et al. 2018). The SBMT has been applied to data from numerous spacecraft missions after first being developed to analyze data from NEAR’s exploration of the asteroid Eros (Kahn et al. 2011). Since then, the SBMT has been used by several flight mission teams, including Dawn (Buczkowski et al. 2012; Blewett et al. 2014; Ruesch et al. 2014; Scully et al. 2014), Rosetta (Besse et al. 2014; Leon-Dasi et al. 2021), OSIRIS-REx (Barnouin et al. 2019; Walsh et al. 2019), and Hayabusa2 (Hirata et al. 2019; Michikami et al. 2019).

**Fig. 1:**
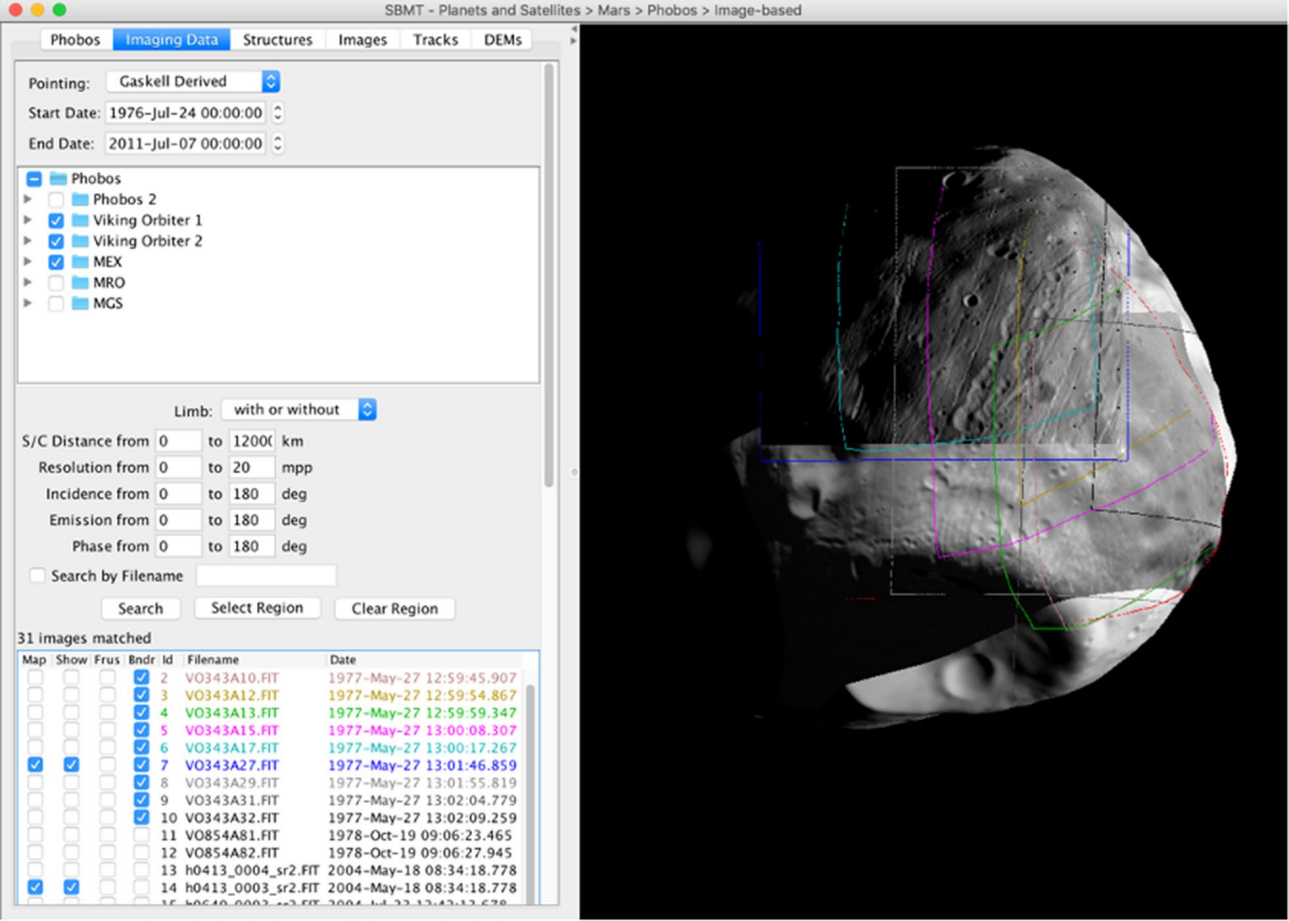
View of Phobos shape model in the SBMT. An HRSC image with a Viking Orbiter image overlain are shown on the shape model, with lighting simulated to match the Viking image. Colored boundaries indicate a subset of additional images available but not visualized on the body

The current publicly accessible version of the SBMT includes key datasets for Phobos and Deimos that were obtained or produced from previous spacecraft missions (Ernst et al., submitted to Earth Planets and Space). To be included in the SBMT, previous images were required to contain Phobos or Deimos with at least 10 pixels across the body and not be completely saturated. Images of Phobos and Deimos came from the Viking Orbiter 1 and 2 Visible Imaging Subsystem (VIS) cameras (Duxbury 1978; Veverka 1978), the Phobos 2 VSK (Murchie and Erard 1996), the MGS Mars Orbiter Camera (MOC) (Thomas et al. 2000), the MRO High Resolution Imaging Science Experiment (HiRISE) (Thomas et al. 2011) and Compact Reconnaissance Imaging Spectrometer for Mars (CRISM) (Fraeman et al. 2012), and the Mars Express High Resolution Stereo Camera (HRSC) (Wählisch et al. 2010). Two altimetry tracks taken during one Phobos flyby were also included from the MGS Mars Orbital Laser Altimeter (MOLA) (Banerdt and Neumann 1999). The section on availability of data at the end of this article provides information of the spacecraft data archives for these missions, at NASA’s Planetary Data System (PDS) and ESA’s Planetary Science Archive (PSA) (Besse et al. 2018). In the SBMT, users can search for, visualize, analyze, and compare these previous datasets, as depicted in Fig. 1. By combining these thousands of images from multiple spacecraft instruments, illumination conditions, and viewing geometries, high-resolution shape models of Phobos and Deimos have been created (Ernst et al. 2015; Ernst et al., submitted to Earth Planets and Space) utilizing stereophotoclinometry techniques (Gaskell et al. 2008). These shape models are available in the SBMT, with resolutions up to just over 3 million plates.

In this work, we adapt the SBMT to a gamma-ray and neutron spectroscopy investigation for the first time. This advancement will lay the ground-work for MEGANE’s investigation of Phobos and also provide a means to enable MEGANE’s measurements to be compared to previous datasets of Phobos and to support cross-instrument investigations once MMX data are acquired. Here, we present the fundamental theory of applying the irregular shape of the body and its proximity to Mars to interpretation of MEGANE’s gamma-ray and neutron spectroscopy data. We then apply the results to the current low-altitude operations plan of the MMX mission, in order to evaluate the measurements that will be made by MEGANE of Phobos during the MMX mission.

## Application of the MEGANE footprint to Phobos

### Spherical geometry

**Fig. 2:**
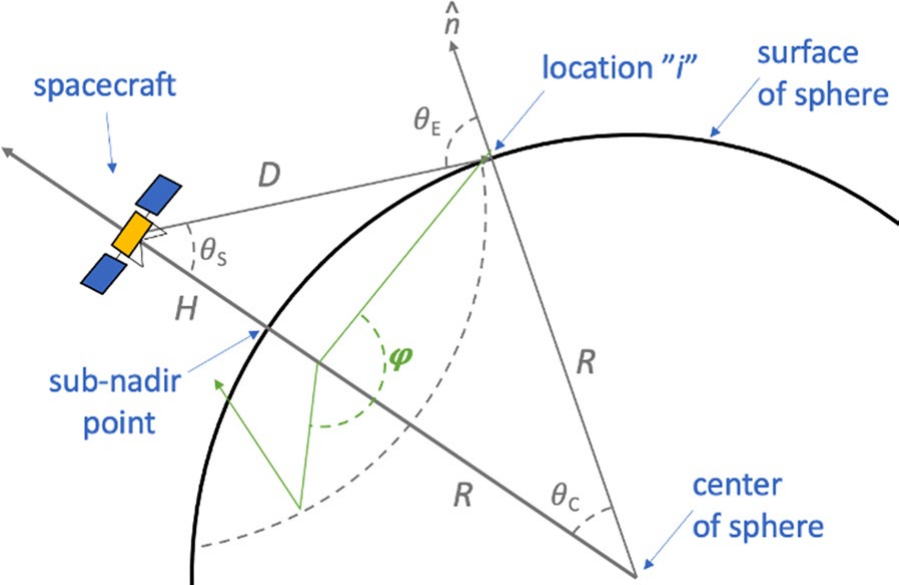
Relevant geometrical parameters for gamma-ray and neutron transport from spherical planetary objects. Adapted with modification from Figure B1 of Prettyman et al. (2006)

Gamma-ray and neutron emissions from planetary objects are a consequence of galactic-cosmic-ray (GCR) induced nuclear spallation reactions, as well as the decay of naturally radioactive elements (e.g., ^40^ K, ^232^Th, and ^238^U). On spherical objects, the planetary surface is uniformly illuminated by GCRs, and the resulting gamma rays and neutrons are emitted from the surface with an angular dependence that is proportion to (cos*θ*_E_)^1/2^ (Lawrence et al. 2006). All geometric parameters presented in this section, including *θ*_E_, are based on the formalism of Prettyman et al. (2006) and defined in Fig. 2.

The total gamma-ray or neutron flux at an orbiting spacecraft is a sum of all of the measured emissions from each individual location *i* across the viewable surface, weighted by the distance from the spacecraft to that point (*D*_*i*_) and the emission-angle dependence of (cos*θ*_E_)^1/2^. For a spherical object, the viewable surface extends from *θ*_S_ = 0, the sub-nadir point to the horizon (*θ*_S_^max^), whose angle is calculated as:

1 $$\theta_{S}^{\max } \left( H \right) = \arcsin \left[ {\frac{R}{{\left( {R + H} \right)}}} \right],$$

where *R* is the radius of the sphere and *H* is the altitude of the spacecraft (above the sub-nadir point).

Calculating the gamma-ray flux measured by the spacecraft begins by segmenting the surface into individual gamma-ray and neutron-emitting facets. For a spherical object, each of these facets is at location *i* and has an area *A*_*i*_, calculated as:

2 $$A_{i} = R^{2} d\omega_{c} d\phi ,$$

where ω_c_ is cos*θ*_c_, and *ϕ* is the azimuthal angle (see Fig. 2). Likewise, ω_E_ is cos*θ*_E_. The gamma-ray or neutron current *J*(ω_E_) from each of these facets is calculated using radiation transport modeling tools like MCNPX (e.g., McKinney et al. 2006; Prettyman et al. 2006) and Geant4 (e.g., Peplowski 2018; Mesick et al. 2018). The number of gamma rays or neutrons emitted from facet at *i* is denoted *j*_γ_(ω_E_, *ϕ*_*E*_) or *j*_n_(ω_E_, *ϕ*_*E*_), respectively. For spherical surfaces, *j*_γ_(ω_E_, *ϕ*_*E*_) and *j*_n_(ω_E_, *ϕ*_*E*_) are independent of azimuthal angle and can be derived from *J*(ω_E_) values as:

3 $$j_{\gamma } \left( {\omega_{E} ,\varphi_{E} } \right) = \frac{{J_{\gamma } \left( {\omega_{E} } \right)}}{2\pi },$$

4 $$j_{n} \left( {\omega_{E} ,\varphi_{E} } \right) = \frac{{J_{n} \left( {\omega_{E} } \right)}}{2\pi },$$

where *J*_*γ*_(ω_E_) and *J*_*n*_(ω_E_) are the model-provided gamma-ray and neutron surface currents, respectively.

The number of gamma rays per unit area at the spacecraft (*N*_γ_) can then be calculated as the sum of all of the contributions from each visible facet as:

5 $$N_{\gamma } = \mathop \sum \limits_{i} \left[ {\frac{{A_{i} \left( {R,\omega_{c} ,\phi } \right)J_{i} \left( {\omega_{E} } \right)}}{{2\pi D_{i}^{2} }}} \right].$$

The number of neutrons at the spacecraft can be similarly calculated, but requires corrections for neutron decay (neutron mean life is ~ 880 s) and the fact that neutrons travel on ballistic trajectories. Details of these corrections are provided by Feldman et al. (1989).

### Application to irregularly shaped objects

**Fig. 3:**
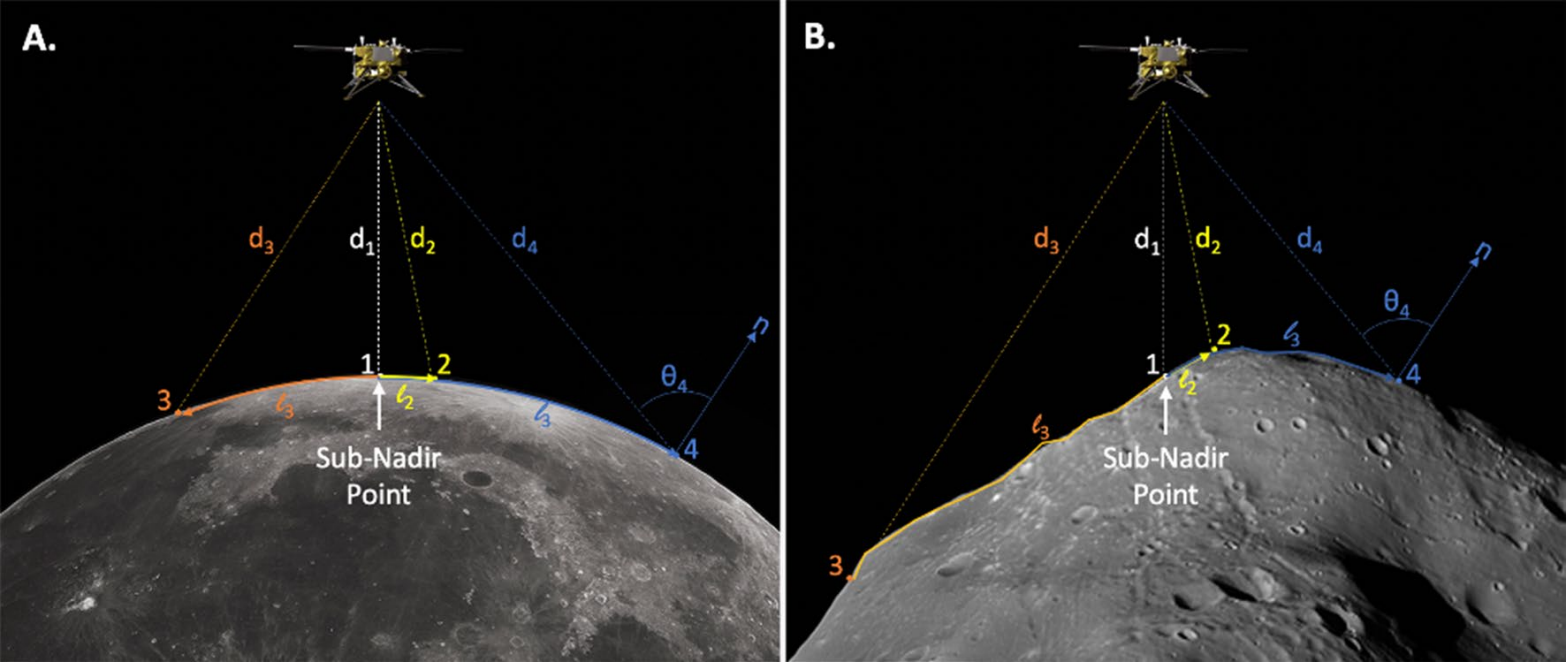
An illustration of some of the geometric differences between nuclear spectroscopy observations of **A** spherical and **B** irregular solar system objects

For spherical objects like planets or large moons, the only object-specific geometric information that is required to solve Eq. 5 is the radius *R*. All other information stems from the use of spherical geometry (e.g., Fig. 2) and knowledge of the spacecraft location. A number of complications arise when considering a nuclear spectroscopy measurement of an irregularly shaped object like an asteroid or a small moon. For example:

1. The sub-nadir point is not necessarily the closest point to the spacecraft (e.g., Point 1 vs. Point 2; Fig. 3),
2. Points with *θ*_S_ < *θ*_S_^max^ are not necessarily in the field of view of the spacecraft due to intervening topography (e.g., Point 3; Fig. 3).
3. The emission angle *θ*_E_ is not related to *θ*_S_, as the surface-normal vector varies for each facet (e.g., Point 4; Fig. 3),

These are just some examples of the complications associated with irregular objects, and they highlight the need for precise knowledge of the three-dimensional shape of the object, like that provided by the shape models used by the SBMT.

Most of the mathematical relations detailed in the “Spherical geometry” section no longer apply for irregularly shaped objects such as Phobos and Deimos. First, the portion of the surface within the field of view of the spacecraft can no longer be calculated using Eq. 1, but instead requires ray-tracing between the spacecraft and each location (facet) on a three-dimensional shape model of the object. Second, Eq. 2 cannot be used to determine the area of each facet within the spacecraft field of view, again because the shape cannot be described in spherical coordinates. Fortunately, shape models inherently describe the shape of the object in terms of facets, and these facets can be adopted unaltered for the nuclear spectroscopy calculations so long as the area of the facet and the direction of its surface-normal vector are known. Finally, the surface-normal vector of each facet, required to determine the emission angle *θ*_E_, must also be determined from the shape model. These quantities are calculated by the SBMT for each facet on Phobos and used in place of the spherical geometry values to solve Eq. 5. In the next section, we examine the fidelity of the shape model needed to be applied to MEGANE applications.

**Fig. 4:**
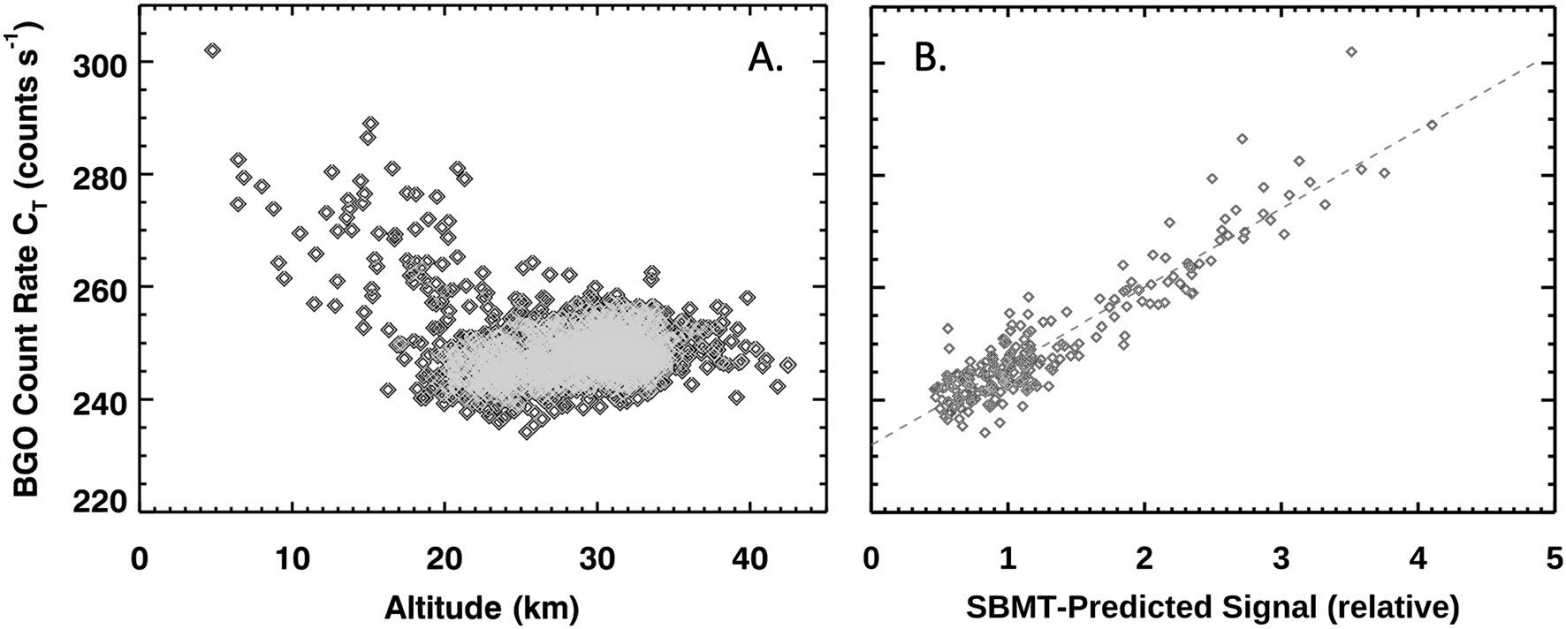
**A** NEAR-GRS-measured gamma-ray rate versus altitude. Altitude is measured from the spacecraft to the sub-nadir point on asteroid 433 Eros. **B** NEAR-GRS-measured versus predicted gamma-ray count rates. Predicted values were derived using a shape model of Eros and Eq. 5. The red-dashed line highlights the linear relationship between the measurements and predictions, validating the approach for calculating gamma-ray fluxes from shape models

We validated Eq. 5 using data collected by the GRS on the Near Earth Asteroid Rendezvous (NEAR) spacecraft. NEAR orbited the asteroid 433 Eros, whose irregular shape (~ 34.4 × 11.2 × 11.2 km) makes it an excellent test of the application of Eq. 5. Peplowski (2016) reported NEAR GRS count rate data and noted that the poor correlation between count rate and altitude (Fig. 4A) could result from Eros’ highly irregular shape. We calculated the gamma-ray flux at NEAR (Eq. 5) using an Eros shape model (Gaskell 2008), along with the spacecraft ephemeris (altitude, latitude, longitude) for each NEAR GRS measurement. The resulting values, which are not corrected for cosmic ray flux, detector area, or detection efficiency, are shown in relative units in Fig. 4B. The excellent linear correlation between the measured and calculated count rates, highlighted by the dashed red line (Fig. 4B), provides real-world validation of our model for calculating gamma-ray fluxes from shape models.

### Examination of shape model resolution

An important item to examine for computing the MEGANE footprint is the resolution required for the Phobos shape model. Higher resolution shape models capture finer-scale topographic features but also increase the computational time required for the calculations. Currently in the SBMT, the shape model produced by Ernst et al., submitted to Earth Planets and Space is available in four standard resolutions: 49,152 plates, 196,608 plates, 786,432 plates, and 3,145,728 plates. The MEGANE footprint on the surface of Phobos is a function of the viewable surface as described in the “Application to irregularly shaped objects” section. Thus, the area of the surface contributing to the MEGANE signal can be quite large for orbital measurements, including covering nearly a hemisphere of the body at any given time, and small-scale topographic features are expected to produce only negligible contributions to the overall MEGANE signal. Consequently, it is anticipated that the 49,152-plate Phobos shape model is sufficient for MEGANE’s calculations and that the use of the higher-resolution Phobos shape models in SBMT are not required, but it is worthwhile to evaluate this expectation.

**Fig. 5:**
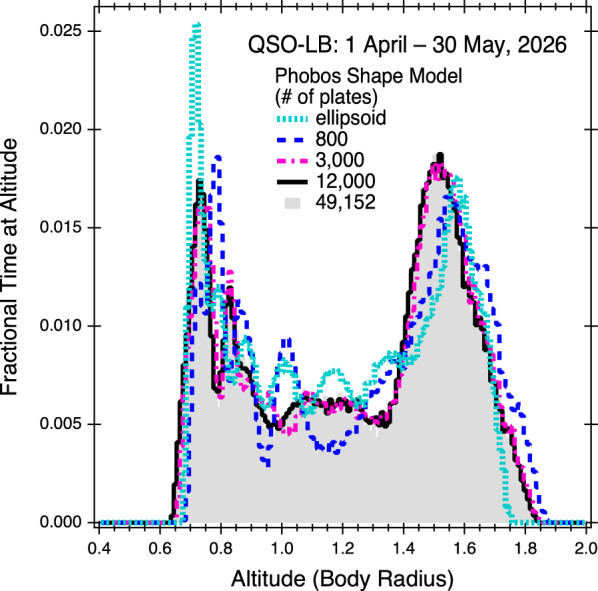
MMX spacecraft altitude calculated for the same operational period but using different resolution shape models. The altitude is plotted in units of body radius, which for any given time is calculated as the spacecraft height above the sub-nadir point (H) divided by the distance from the center of Phobos to that sub-nadir point (R)

The first evaluation was to examine the effect of the resolution of the shape model on the calculation of the spacecraft’s altitude. MEGANE’s measurements are highly sensitive to spacecraft altitude, as shown in the “Spherical geometry” section. As a rule of thumb, the spatial resolution of a gamma-ray or neutron investigation is proportional to the altitude, i.e., an altitude of 10 km yields a full-width at half-maximum spatial resolution of ~ 10 km. For this evaluation, the Ernst et al., submitted to Earth Planets and Space derived shape model with 49,152 plates was used as a basis to create lower resolution, 800-, 3000-, and 12,000-plate shape models of Phobos. A 49,000-plate ellipsoid based on Phobos IAU radius values of 13.0, 11.4, and 9.1 km (Archinal et al. 2018) was also produced. These different resolution shape models were then used along with a MMX trajectory file that covers the planned operations about Phobos from 1 April to 30 May 2026 (Nakamura et al. 2021) to calculate the spacecraft altitude over this duration. Figure 5 shows the altitude calculation results, which show that using the ellipsoid that does not account for Phobos’ irregular shape results in altitude calculations that differ from when a higher-fidelity Phobos shape model is used. Additionally, the Phobos shape model with only 800 plates results in altitude calculations that differ from the higher-resolution shape models, demonstrating that the 800-plate model is insufficient for calculating the MEGANE footprint on Phobos. The 3000- and 12,000-plate shape models yield similar altitude histogram results to the 49,152-plate model (Fig. 5), demonstrating a shape model resolution of 3000 plates or better is sufficient for MEGANE’s altitude calculations.

**Fig. 6:**
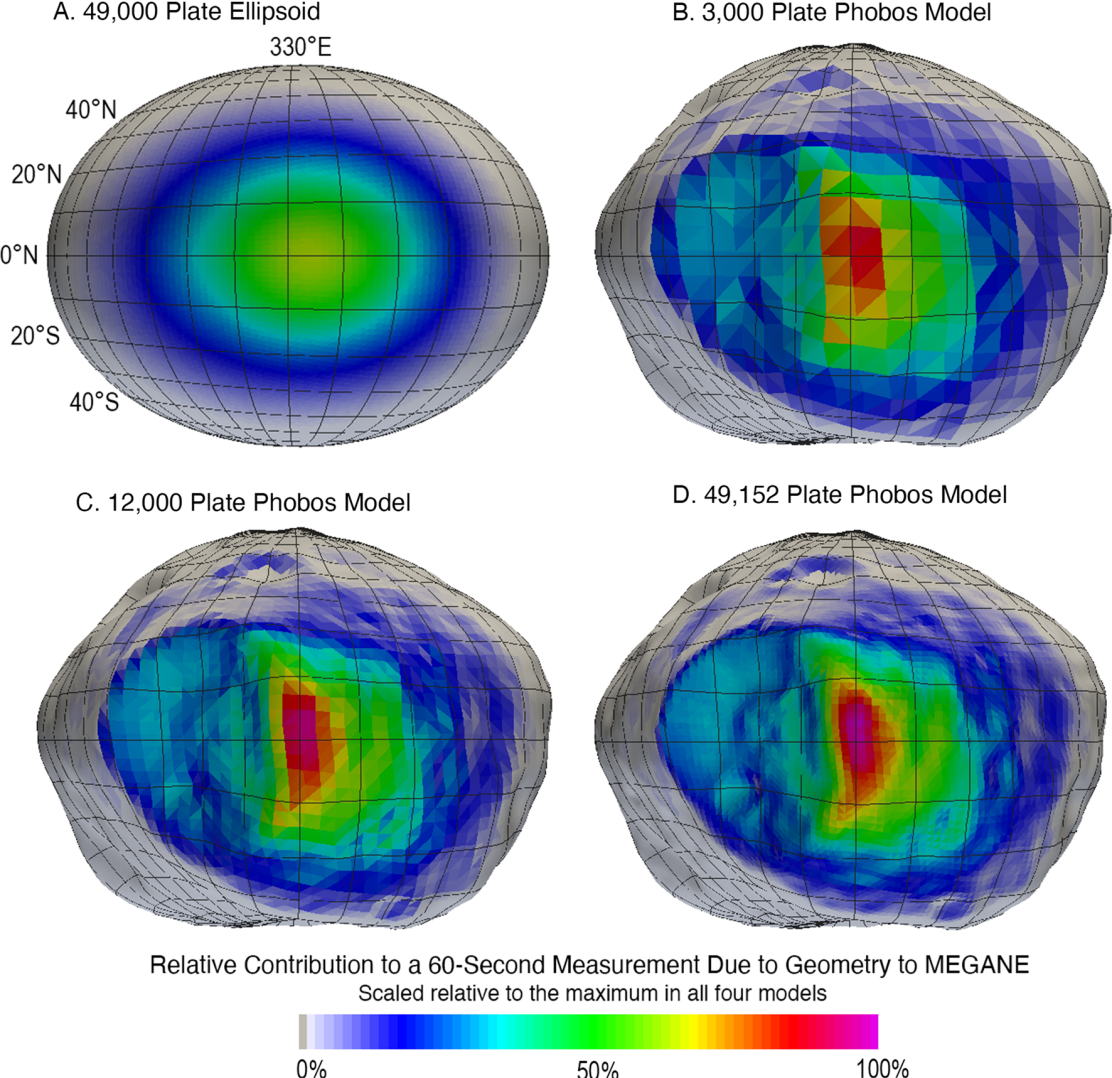
Comparison of different resolution shape models, **A** 49,000 plate ellipsoid and **B** 3000, **C** 12,000, and **D** 49,152 plate Phobos models, used to calculate the relative contribution to a 60-s MEGANE measurement due to the geometry between the MMX spacecraft and each location on Phobos’ surface. The 60-s example shown uses MMX trajectory information for 1 April 2026, operating in QSO-LB, with a sub-nadir point of 0.1°N and 330.0°E and an altitude of 0.7 body radius

A second evaluation was to examine the implementation of the MEGANE footprint described in the “Spherical geometry” and the “Application to irregularly shaped objects” sections to different resolution Phobos shape models, and those results are shown in Fig. 6. Including the irregular shape of Phobos affects the relative contributions to the MEGANE measurement of different surface locations. Even utilizing the low-resolution, 3000-plate Phobos shape model results in noticeable differences in interpreting which portions of Phobos’ surface are contributing most strongly in the 60-s footprint in comparison to the ellipsoid shown in Fig. 6. The higher-resolution shape models of 12,000 and 49,152 plates provide finer details of the relative contributions of different surface locations, but those differences are small in comparison to the total spatial extent of the 60-s footprint. Overall, the large-scale pattern of the relative contribution to a MEGANE measurement is similar when using Phobos shape models with ≥ 3000 plates. This conclusion is further discussed and supported in the “MEGANE mapping of Phobos” section with mapping Phobos’ blue unit.

These evaluations indicate that a shape model of at least 3000 plates is required for interpretation of MEGANE measurements. Thus, the 49,152-plate Phobos shape model in the SBMT is more than sufficient to include the effects of Phobos topography on MEGANE’s resulting measurements.

### Viewable sky considerations for Phobos

**Fig. 7:**
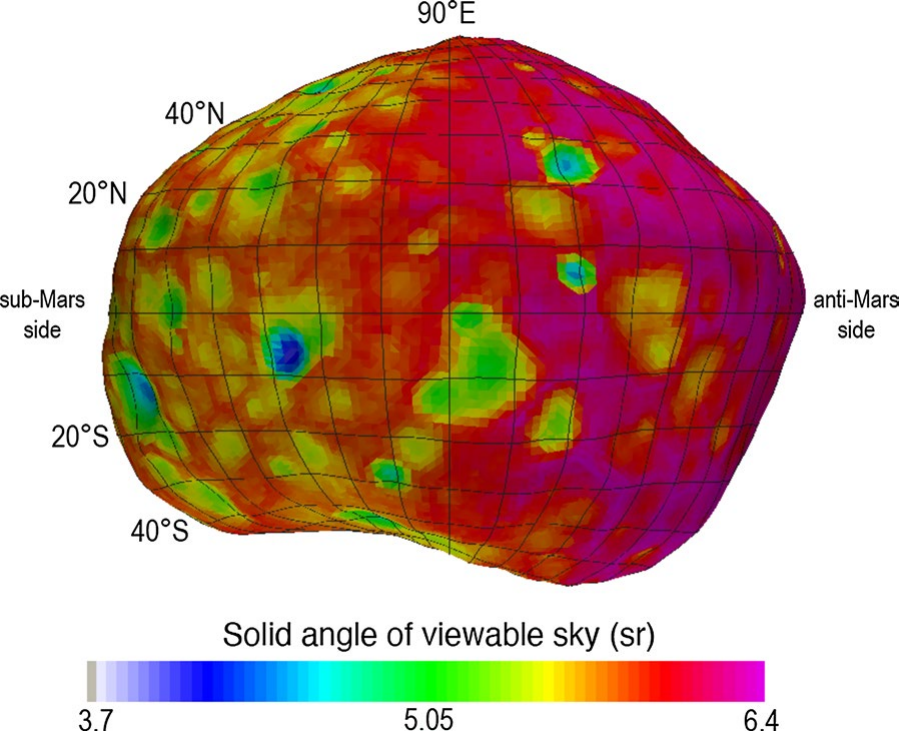
Solid angle of the viewable sky (Ω_sky_), with the eastern hemisphere of Phobos shown as an example. The effects of topography are noticeable, along with lower viewable sky values on the sub-Mars side due to Mars obstructing the view of the sky

The SBMT is also valuable for examining gamma-ray and neutron production on Phobos, as Phobos’ irregular shape has implications for the surface-incidence GCR rate at the surface. Local topography like crater walls can reduce the field of view to space, meaning that these locations will experience a reduced flux of GCRs and thus have lower gamma-ray and neutron emissions. Additionally, Mars blocks an appreciable fraction of the sky on the Mars-facing hemisphere. To quantify this effect, we used the Phobos shape model to calculate the solid angle of the viewable sky (Ω_sky_) at each location on Phobos’ surface (Fig. 7). Here Ω_sky_ quantifies the view to open space, meaning that the portion of the sky subtended by Mars is not included.

The maximum (unobstructed) view to space for an observer on a spherical surface is 2π sr (Ω_sky_ ~ 6.28 sr). While a few locally elevated points on Phobos have an unobstructed view to space that exceeds a hemispherical view, up to 6.38 sr, we found that on Phobos, Ω_sky_ can be as low as 3.7 sr, 57% of the maximum value, within the deepest craters. As a result, the gamma-ray and neutron emissions from within these craters will be reduced by up to 43%. If uncorrected for, this phenomenon would be mistaken for variations in surface composition during analysis of MEGANE data.

Phobos orbits Mars with a mean orbit radius of 9,376 km (2.76 Mars radii), in a tidally locked configuration with a small libration amplitude of 1.09° (Oberst et al. 2014); the sub-Mars point is defined as 0º longitude. Accordingly, Mars’ location in the sky is essentially fixed, and it is observable only from the sub-Mars hemisphere. Our Ω_sky_ calculation includes the effect of Mars, which subtends 0.42 sr (6.7% of the sky) at the sub-Mars point. The value at any given position depends on the position and local topography. The effect of Mars is visible in Fig. 7 as a hemispherical dichotomy wherein Ω_sky_ is lower on the sub-Mars side of Phobos. The consequence is lower cosmic-ray exposure on the Mars-facing hemisphere, and a corresponding lower gamma-ray and neutron flux.

The Ω_sky_ map provides a correction to Eq. 5. As noted in the “Spherical geometry” section, the gamma-ray and neutron current at the surface of Phobos is calculated using radiation transport models. Those models use a spherical target object, which greatly simplifies the setup of the model and minimizes the computational time required by leveraging the spherical symmetry of the geometry. Yet this approach introduces an assumption, that Ω_sky_ is always 2π sr. Corrections to Eqs. 3 and 4 are therefore needed to account for the actual Ω_sky_ value. These corrected equations take the form:

6 $$j_{\gamma } \left( {\omega_{E} ,\varphi_{E} } \right) = \frac{{J_{\gamma } \left( {\omega_{E} } \right)}}{2\pi }\frac{{{\Omega }_{sky} }}{2\pi },$$

and

7 $$j_{n} \left( {\omega_{E} ,\varphi_{E} } \right) = \frac{{J_{n} \left( {\omega_{E} } \right)}}{2\pi }\frac{{{\Omega }_{sky} }}{2\pi }.$$

These corrections shift the computational resources associated with the irregular shape of Phobos from the radiation transport models to the SBMT, which is better suited for this challenge.

## Results

### MEGANE mapping of Phobos

**Fig. 8:**
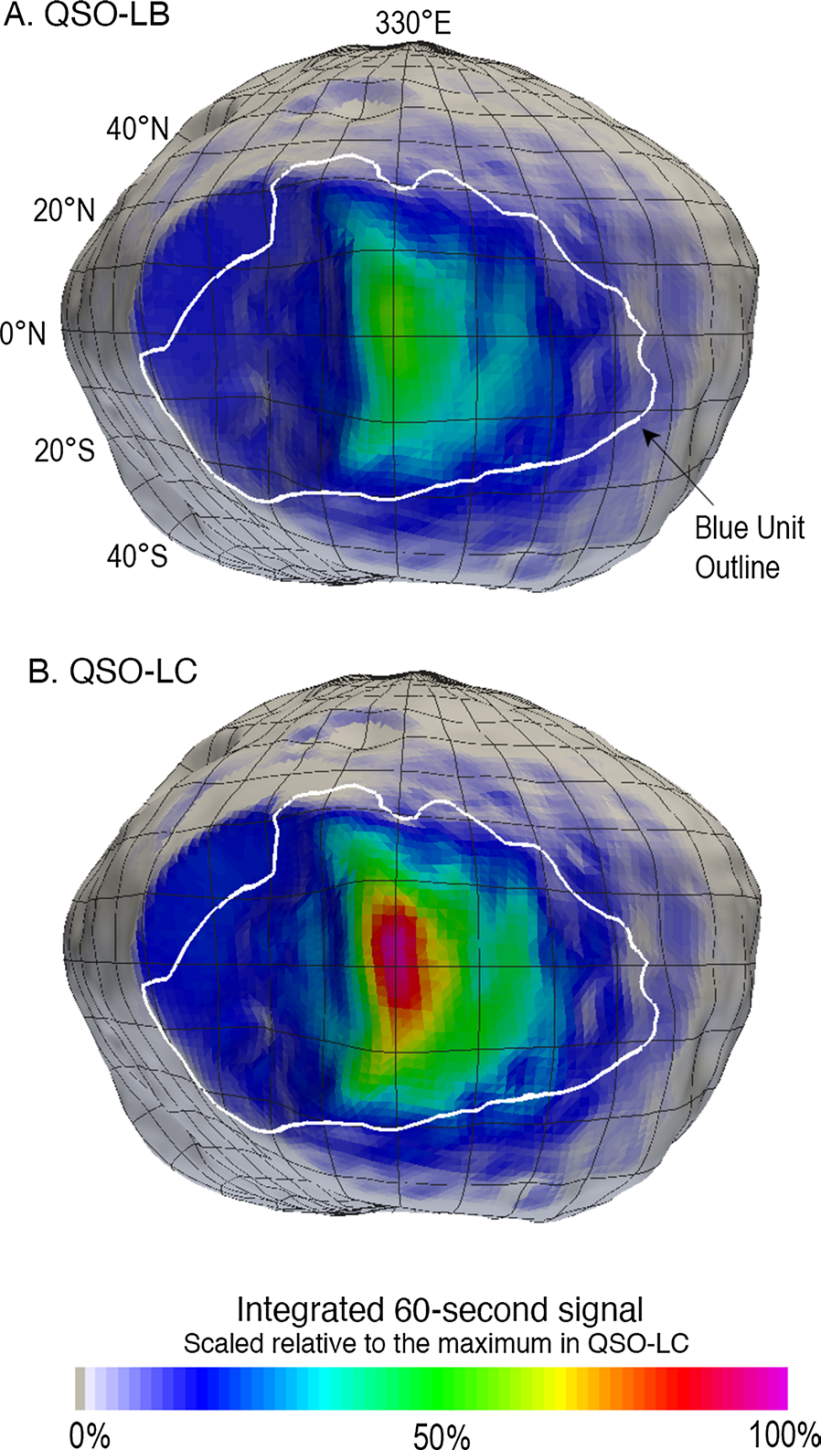
Comparison of a 60-s MEGANE integrated signal acquired in **A** QSO-LB on 1 April 2026 at an altitude of 0.7 body radius and **B** QSO-LC on 1 June 2026 at an altitude of 0.5 body radius. The lower-altitude QSO-LC provides a higher integrated total signal for MEGANE

The expected mapping of Phobos by MEGANE during the MMX mission can be examined by applying all of the methods in the “Application of the MEGANE footprint to Phobos” section that cumulate in Eqs. 6 and 7. The MMX mission baseline plan used for MEGANE’s Critical Design Review (CDR) in June 2021 included low-altitude operations that are well-suited to MEGANE measurements during the time period of 1 April to 15 July 2026 (Nakamura et al. 2021). The specific details of the MMX operational plans are likely to continually evolve as the mission progresses, but these CDR baseline plans are illustrative of how MEGANE’s science measurements of Phobos will be accomplished. There are two main quasi-satellite orbits (QSO) that are being considered during this time period: QSO-LB, with its short axis being roughly 22 km from Phobos center, and the slightly lower altitude QSO-LC, with its short axis roughly 20 km from Phobos center. Figure 8 compares the 60-s footprint for QSO-LB and QSO-LC selected from those QSO trajectory files at a time to be similarly positioned over Phobos’ blue unit (Murchie and Erard 1996).

One of MEGANE’s science requirements is to determine the composition of Phobos’ blue unit (Lawrence et al. 2019). The measurements from both QSO-LB and QSO-LC are well-suited to measure the blue unit, and, for the footprints shown in Fig. 8, 73% of MEGANE’s signal is located within the blue unit for the QSO-LB example, and 79% of the signal is located within the blue unit for the QSO-LC example. In addition to sampling the blue unit, the lower altitude QSO-LC provides the opportunity for higher signal contribution in general from within the blue unit than that obtained during QSO-LB.

The MMX plan devotes the 105-day period of 1 April to 15 July 2026 to acquiring the lowest altitude measurements of Phobos prior to landing and sample collection (Nakamura et al. 2021). One option under consideration is spending 60 days in QSO-LB followed by 45 days in QSO-LC. Here we show examples from this option, as it enables a comparison between the scientific measurements that MEGANE can make in each of the two QSOs. We assume that the MMX spacecraft always points nadir for the mapping examples shown in this study, even though thermal constraints, off-nadir operations of other instruments, and the need to point to the Earth for communications and to transmit data make this an upper bound on the total nadir pointing time (Nakamura et al. 2021). Nevertheless, the nadir-pointed assumption is useful for mapping the *relative* contributions from different portions of Phobos’ surface to the MEGANE integrated signal. The sub-spacecraft longitude repeats on a period of 4.4 h in QSO-LB and 3.96 h in QSO-LC. Over the 105-day duration of low-altitude measurements, all longitudes are covered many times. Similarly, the spacecraft is over the sunlit Phobos surface for a period of 5.2 h in QSO-LB and 4.1 h for QSO-LC, followed by similar durations over the nightside surface. These day-night durations are small in comparison to the total 105 days of low-altitude observations. Thus, any systematic off-pointing that occurs due to thermal constraints that limit nighttime observations or Earth communications systematically planned for the nightside will average out when considering mapping timescales greater than or equal to tens of days.

**Fig. 9:**
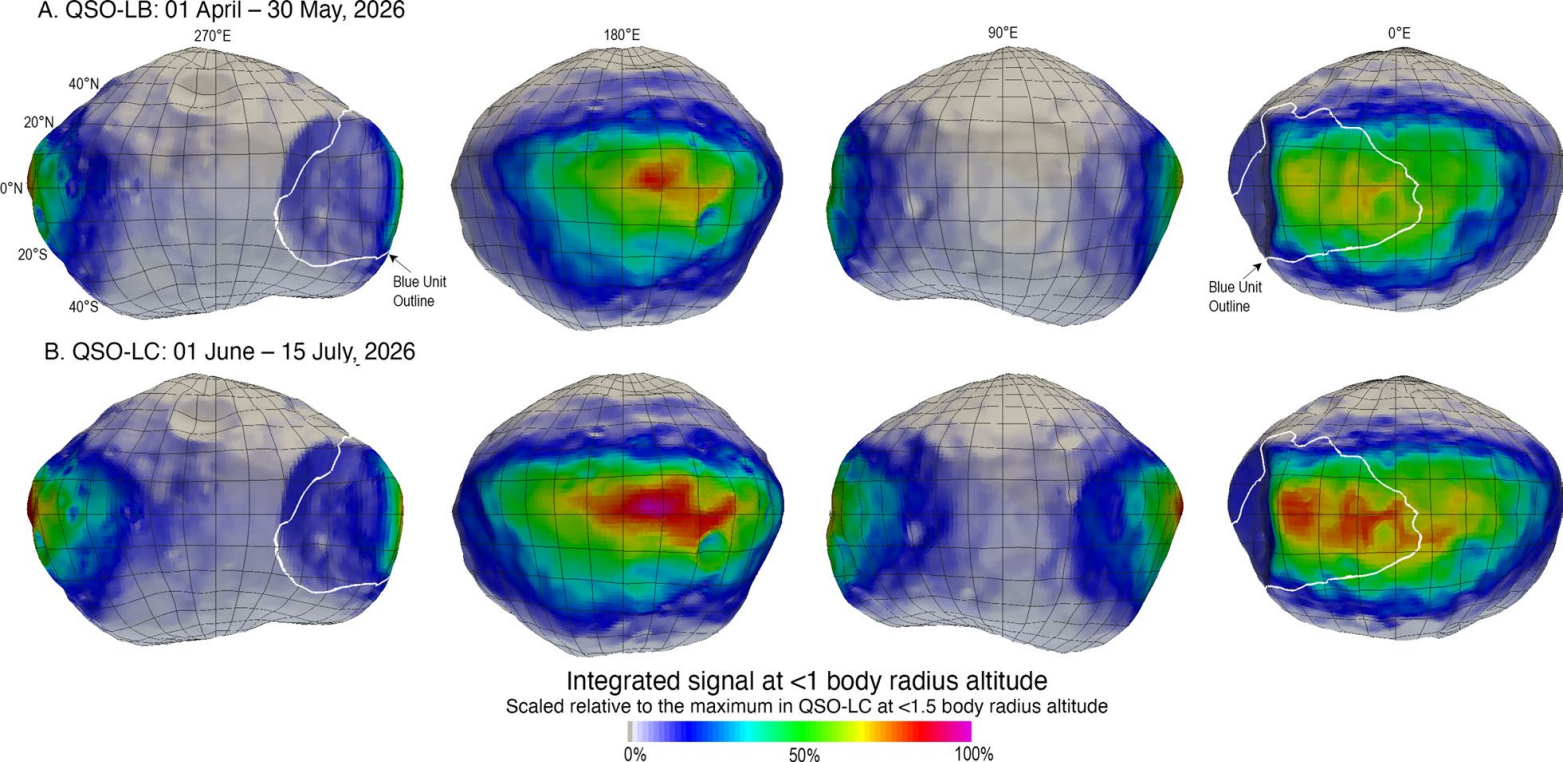
Integrated MEGANE signal from altitudes ≤ 1 body radius, the GRS altitude requirement, for the MMX planned mapping period in **A** QSO-LB and **B** QSO-LC. The color scale depicts the contribution of each surface element to the total MEGANE signal over these durations and uses the same scale as Fig. 10, for comparison to the mapping from ≤ 1.5 body radius, the NS altitude requirement

Analysis of previously flown gamma-ray instruments and their resulting science measurements has shown that cumulative measurements at altitudes ≤ 1 body radius are required to ensure sufficient sensitivity to meet MEGANE’s science measurement objectives (Peplowski 2016). Figure 9 displays the resulting cumulative integrated signal obtained with an altitude of ≤ 1 body radius for 60 days in QSO-LB or 45 days in QSO-LC. The near equatorial paths of both QSO-LB and QSO-LC limit the MEGANE coverage to within roughly 20° latitude of the equator. The coverage is not uniform across all Phobos longitudes as the altitude above the surface varies throughout the QSO, with altitudes ≤ 1 body radius near the sub-Mars and anti-Mars hemispheres but > 1 body radius for the hemispheres centered on 90°E and 270°E. The longitude coverage is more extensive for the QSO-LC option, though is not complete in either QSO option (Nakamura et al. 2021).

Both QSO options provide good coverage of Phobos’ blue unit, whose general location is outlined in Fig. 9. Overall, for the QSO-LB mapping shown in Fig. 8, 31.6% of the QSO is spent at altitudes ≤ 1 body radius, and 6.8% of the time is spent at ≤ 1 body radius and with ≥ 50% of the MEGANE signal originating from the blue unit. MEGANE’s GRS science requirements include at least 240 h of cumulative measurements at ≤ 1 body radius with MEGANE within 10° of nadir pointed and at least 40 h of that time at ≤ 1 body-radius altitude with the majority of the MEGANE signal from the blue unit. These percentages show that if the 240-h accumulated measurement time conditions are met, the required coverage of the blue unit will also be achieved by measurements in QSO-LB, even accounting for effects due to Phobos’ irregular shape. Similarly, for the QSO-LC mapping in Fig. 9, 46.6% of the QSO is spent at altitudes ≤ 1 body radius and 9.4% is spent at ≤ 1 body radius and with ≥ 50% of the MEGANE signal originating from the blue unit. Thus, QSO-LC is also well-suited to meet the required coverage of the blue unit if the overall 240-h total accumulated measurement time is achieved.

As a further test of the adequacy of the resolution of the shape model used in our approach, calculations of MEGANE’s blue unit coverage were performed using both the lower resolution 3,000-plate shape model and the 49,152-plate shape model, as shown in Fig. 6. The fractional amount of time when the MEGANE signal was located within the blue unit using the QSO-LB case above differed by only 0.008% between the two calculations (6.797% for the 3000-plate model versus 6.805% for the 49,152-plate model). This comparison is further confirmation that the 49,152-plate resolution of the shape model being used for MEGANE is more than sufficient for measuring features of interest on Phobos’ surface, such as the blue unit.

**Fig. 10:**
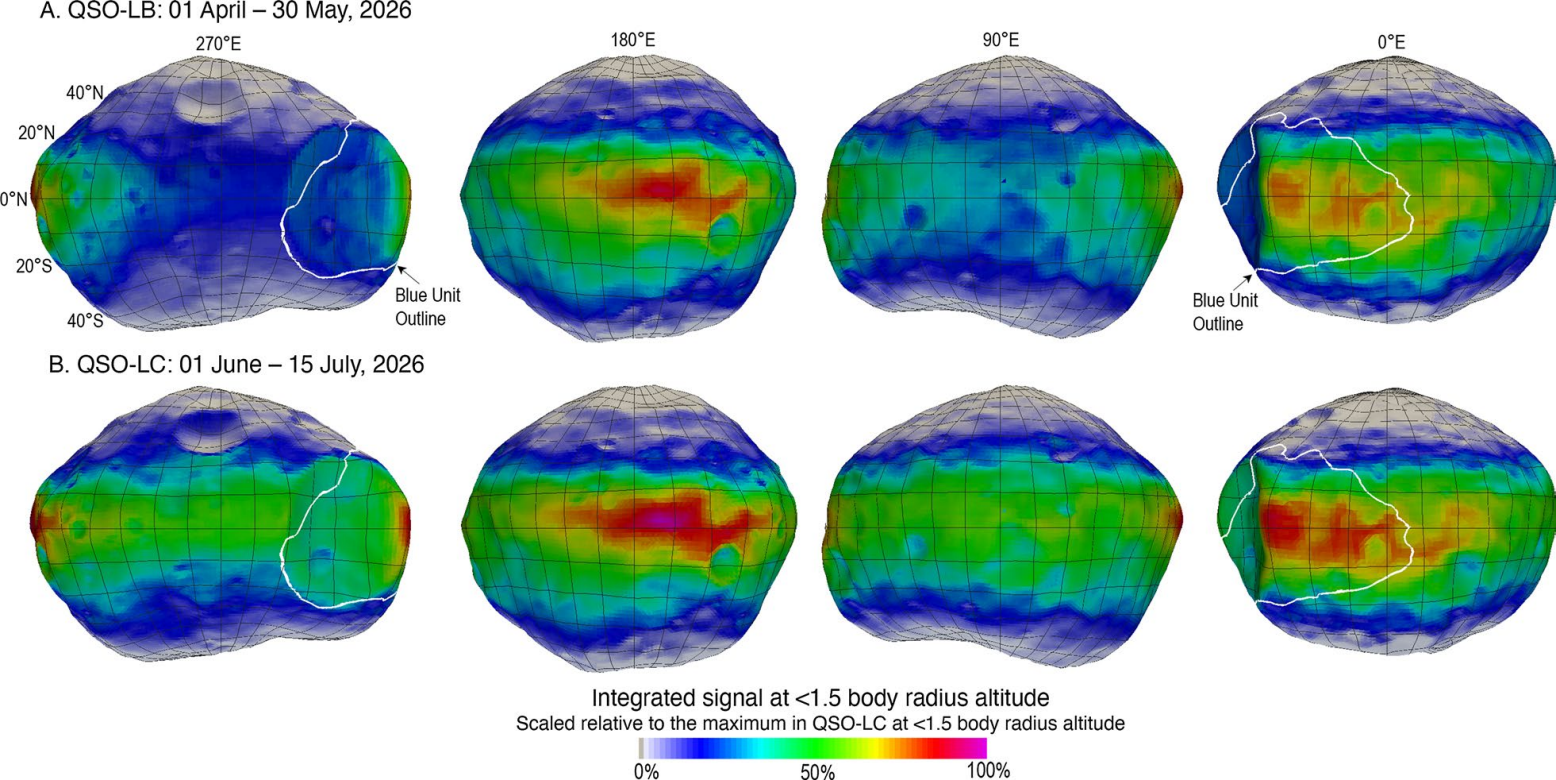
Integrated MEGANE signal from altitudes ≤ 1.5 body radius, the NS altitude requirement, for the MMX planned mapping period in **A** QSO-LB and **B** QSO-LC. The color scale depicts the contribution of each surface element to the total MEGANE signal over these durations and uses the same scale as Fig. 9, for comparison to the mapping from ≤ 1 body radius, the GRS altitude requirement

While MEGANE GRS measurements require altitudes ≤ 1 body radius, MEGANE NS measurements with sufficient signal to background resolution can be achieved at higher altitudes, with the MEGANE requirement being that NS measurements are acquired at ≤ 1.5 body radius (Lawrence et al. 2019). Thus, the NS measurement altitude is not a driving requirement for MEGANE operations; if the GRS requirement is met, the NS requirement is met as well. However, the higher altitude for NS measurements noticeably affects the mapping coverage for Phobos, as shown in Fig. 10. In comparison to the ≤ 1 body radius mapping coverage in Fig. 9, the ≤ 1.5 body radius mapping provides increased coverage of Phobos longitudes. In particular, QSO-LB spends 68% of its time at an altitude ≤ 1.5 body radius while QSO-LC spends 100% of its time below an altitude of 1.5 body radius (Nakamura et al. 2021). Thus, QSO-LC provides an excellent opportunity for MEGANE NS measurements across all longitudes of Phobos, as shown in Fig. 10B.

MEGANE requirements include measuring the blue unit of Phobos, because that is a spectral unit currently known on Phobos from the limited, largely opportunistic, spacecraft investigations to date. As the first spacecraft dedicated to the exploration of Phobos, the multi-instrument observations by MMX will greatly increase the resolution and fidelity of our knowledge of Phobos’ surface. As such, it is reasonable to expect that MMX data will result in mapping new units and features on the surface of Phobos. The blue unit outline shown in Figs. 8, 9, 10 was mapped using the SBMT and a color composite of Phobos formed from Mars Reconnaissance Orbiter images (Thomas et al. 2011). A combination within the SBMT of unit-mapping and a means to evaluate which MEGANE 60-s footprints fall within the outlined unit will be applied to Phobos’ blue unit when data are returned by the MMX mission in 2026. Importantly, this approach can be similarly applied to any new units identified during the MMX mission. Developing this footprint-evaluation capability in the SBMT is thus highly worthwhile, both to measure the blue unit and to be ready to contribute to new discoveries during the MMX mission.

### Comparisons with other martian moons datasets

Over the past four decades, an armada of spacecraft have imaged Phobos and Deimos, thereby providing key datasets of these bodies. MMX’s dedicated multi-year, multi-instrument mission (Nakamura et al. 2021) to the martian moons is set to transform our understanding of these bodies. The ability to cross-analyze disparate datasets, from MMX and from previous missions, will enable unique Phobos and Deimos science to be performed by the multi-disciplinary MMX team that cannot be achieved using one dataset alone. There can be major challenges inherent in analyzing and cross-correlating the available datasets returned by past Mars-bound spacecraft sent by NASA, ESA, and the USSR and the revolutionary orbital datasets that will be obtained by JAXA’s MMX mission. The data assortment obtained by numerous spacecraft and instruments can be difficult to synthesize and compare, due to complexities of coordinating spacecraft positioning, instrument pointing, data calibration, and data archives. Yet this synthesis is critical for comprehensive analyses of the martian moons. The SBMT is well-suited to addresses these challenges and to be used by the MEGANE and larger MMX team as new MMX datasets of the martian moons are obtained.

Once low-altitude MEGANE data of Phobos are acquired in 2026, the SBMT will be utilized as discussed in the “MEGANE mapping of Phobos” section. The SBMT will be used to aid MEGANE analysis, by taking into account the irregular shape of Phobos and the sky visibility effects on the measurements acquired by MEGANE. Additionally, the SBMT will enable the MEGANE data to be searched, to find the measurements that cover regions of interest, such as the blue unit, to identify those subsets of MEGANE measurement times and combine them to determine compositional differences across MEGANE’s surface. All MEGANE datasets and products will be archived in and publicly available in NASA’s Planetary Data System (PDS), and the final MEGANE compositional maps will also be placed into the SBMT and publicly available, to enable comparisons to other datasets.

Additionally, the SBMT has existing capability to incorporate other MMX datasets, ranging from individual images and measurements to resulting mapping products. The MMX mission has a robust set of instruments that can be used for multi-instrument comparisons and scientific investigations of Phobos’ surface. In addition to MEGANE, these include:

- TENGOO—TElescopic Nadir imager for GeOmOrphology (Kameda et al. 2019), a narrow-angle visible imager
- OROCHI—Optical RadiOmeter composed of CHromatic Imagers (Kameda et al. 2019), seven wide-angle bandpass imagers, from 390 to 950 nm, and a panchromatic imager
- MIRS—MMX InfraRed Spectrometer (Barucci et al. 2020), an imaging spectrometer that covers 0.9–3.6 µm
- LIDAR—LIght Detection and Ranging (Matsumoto et al. 2020), a laser altimeter at 1064 nm.

As data from multiple MMX instruments are acquired, the SBMT will be available to the team and can be used to visualize MEGANE results and also potentially enable cross-instrument comparisons to address the science goals of the mission.

Lastly, while MEGANE measurements of Deimos will require close flybys of roughly 10 km altitude or closer, and it is not clear if that will be possible by the MMX mission (Nakamura et al. 2021), a high-resolution Deimos shape model (Ernst et al., submitted to Earth Planets and Space) along with images from Viking Orbiter, MOC, HiRISE, HRSC, and CRISM are available in the SBMT and can be used by the public and the MMX team. Comparisons between Phobos and Deimos can provide key scientific insight into the origin and formation of the martian moons by showing if the two moons are compositionally similar or rather have differences. The SBMT can help enable such comparisons through using the same tool to examine both bodies.

## Summary and next steps

The irregular shape of Phobos and its proximity to Mars both affect the measurements that MEGANE will obtain during the low-altitude operations of the MMX mission. The SBMT has a strong history of being used to investigate many other irregularly shaped bodies through mapping of images and altimetry products. For the MMX mission, we are furthering the development of the SBMT to include MEGANE’s measurements, which is the first time the SBMT is being applied to a gamma-ray and neutron spectroscopy dataset. Efforts accomplished to date include:

- Implementation and validation of the mathematical equations that relate the effect of topography on MEGANE’s view of the surface and the resulting signals obtained by the instrument aboard the MMX spacecraft.
- Implementation of the effect of topography and the proximity of Mars on the visible sky to include this effect on the GCRs and the effect on the resulting MEGANE measurements.
- Utilization of the current best MMX low-altitude QSO trajectory files to evaluate the relative surface coverage obtained by QSO-LB and QSO-LC and the coverage of Phobos’ blue unit.

With these developments complete, the foundation has been set for the SBMT to be used in preparation for and during MEGANE’s investigation of Phobos. The next steps will involve incorporating these advancements into the SBMT with user-friendly options and to make that version available to the full MMX team. The ability to search on the anticipated MEGANE data within the SBMT is also one of the next priorities for development. Such a search would be used to identify which measurement times should be investigated to determine the composition of the blue unit. Also, having such a search capability is important so that when the MMX mission makes new discoveries of new surface units, the MEGANE team can quickly contribute and evaluate if there are any compositional differences that can be measured from the MEGANE data. Additionally, we will investigate how to apply the SBMT calculations to the analysis of different surface units on Phobos by fully considering the fraction of any given footprint that falls within a given unit of interest. By weighting different measurements according to the percentage of the MEGANE signal in a given unit, we may gain a better understanding of the true end-member compositional differences on the surface of Phobos. Lastly, we welcome the inclusion of other MMX datasets into the SBMT and look forward to the SBMT being a useful resource for the MMX mission and for future investigators who utilize MEGANE’s public PDS archive.

## Data Availability

Data used in this study are available via the Small Body Mapping Tool: https://sbmt.jhuapl.edu/. The original spacecraft data shown in the Small Body Mapping Tool are available at the NASA PDS and ESA PSA: Viking Orbiter:VIS: https://pds-geosciences.wustl.edu/missions/viking/visedr.html Phobos 2:VSK: https://pdssbn.astro.umd.edu/holdings/phb2-m-vsk-2-edr-v1.0/dataset.shtml MGS:MOC: https://pds-geosciences.wustl.edu/missions/mgs/moc.html MEX:HRSC (including SRC): https://www.cosmos.esa.int/web/psa/mars-express MRO:HiRISE: https://pds-imaging.jpl.nasa.gov/portal/mro_mission.html The NASA PDS Image Atlas is an interactive tool that facilitates searches for Phobos and Deimos images from Viking Orbiter, MOC, and HiRISE: https://pds-imaging.jpl.nasa.gov/search/ The ESA PSA has a web search interface that facilities searches for Phobos and Deimos images from HRSC: https://archives.esac.esa.int/psa/

## Abbreviations

CDR: Critical design review
CRISM: Compact Reconnaissance Imaging Spectrometer for Mars
ESA: European Space Agency
GCR: Galactic cosmic ray
GRS: Gamma ray spectrometer
HiRISE: High Resolution Imaging Science Experiment
HRSC: High resolution stereo camera
LIDAR: LIght Detection and Ranging
MEGANE: Mars-moon Exploration with GAmma rays and Neutrons
MEX: Mars Express
MGS: Mars Global Surveyor
MIRS: MMX InfraRed Spectrometer
MMX: Martian Moons eXploration
MOC: Mars Orbiter Camera
MOLA: Mars Orbital Laser Altimeter
NASA: National Aeronautics and Space Administration
NEAR: Near Earth Asteroid Rendezvous
NS: Neutron spectrometer
OROCHI: Optical RadiOmeter composed of CHromatic Imagers
PDS: Planetary Data System
PSA: Planetary Science Archive
QSO: Quasi-satellite orbits
SBMT: Small Body Mapping Tool
TENGOO: TElescopic Nadir imager for GeOmOrphology
VIS: Visible imaging subsystem
